# Supplementary material for: Allele-level visualization of transcription and chromatin by high-throughput imaging
Source: bioRxiv. 2024 Feb 19:2024.02.19.580973. Preprint. [Version 1] doi: 10.1101/2024.02.19.580973 (PMC10962702; doi:10.1101/2024.02.19.580973)
Supplement: Supplement 1 [file NIHPP2024.02.19.580973v1-supplement-1.pdf]

bioRxiv preprint doi: https://doi.org/10.1101/2024.02.19.580973; this version posted February 19, 2024. The copyright holder for this preprint (which was not certified by peer review) is the author/funder. This article is a US Government work. It is not subject to copyright under 17 USC 105 and is also made available for use under a CC0 license.

Supplementary Information

Figure 1: Sequence and location of DNA and RNA probes binding sites

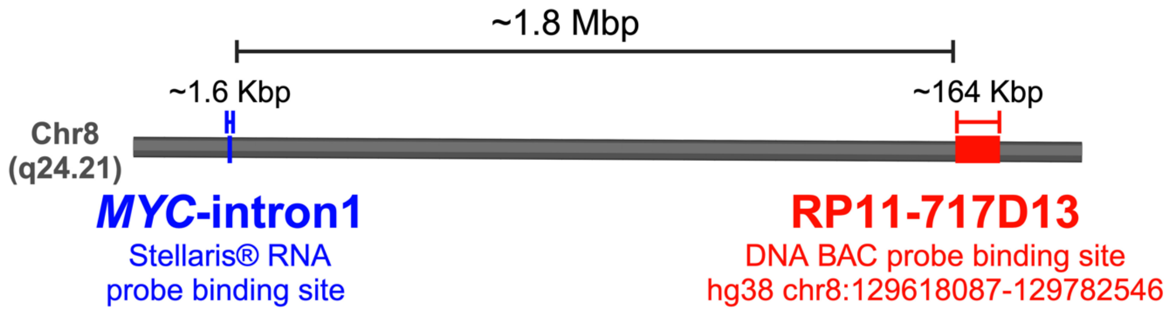

MYC **Stellaris® RNA Probes hybridization sites**

(Intron1, hg38 chr8:127736231-127737854, strand: +, 5' to 3')

gtaagcaccgaagtccacttgccttttaatttatttttttatcacttt**aatgctgagatgagtcgaat**gcctaaatagggtgtcttttctcccattcc
tgcgctattgacacttt**tctcagagtagttatggtaa**ctggggctggggtggggggtaatccagaactggatcg**gggtaaagtgacttgt
caag**atgggagaggagaaggcagagggaaa**acgggaatggtttttaagac**taccctttcgagatttctgccttatgaatatattcacgctg
actcccggcc**ggtcggacattcctgcttta**ttgtgtt**aattgctctctgggttttgg**ggggctgggggttgctttgcggtgggcagaaagc
cc**cttgcatcctgagctccttg**gagtagggaccg**catatcgcctgtgtgagcca**gatcgctccgcagccg**ctgacttgtccccgtctcc
g**ggagggcatttaaatttcggctcaccgcatttctgacagccggagacggacactgcggcgcgtcccgcccgcctgtccccgcggcgatt
ccaa**cccgccctgatccttttaag**aagttggcatttggctttttaaaaagcaataatacaatttaaaacctgggtctctaga**ggtgttaggacg
tggtgttg**ggtaggcgcaggcaggggaaaagggaggcga**ggatgtgtccgattctcctg**gaat**cgttgacttggaaaaaccag**ggc
gaatctccgcacccagccctgactcccctgccgcggccgccctcgggtgtcctcgcgcccgagatgcggaggaactgcgaggagcggg
gctctgggcggttc**cagaacagctgctacccttg**gtggggtggctccgggggaggtatcgcagcggggtctctggcgcagt**tgcatctc
cgtattgagtgc**gaag**ggaggtgcccctattattat**ttgac**accccccttgtatttatgga**ggggtgttaaagcccgcggctgagctcgc
cactccagccggcgagagaaagaagaaaagctggcaaaaggagtgttggacgggggcggtactgggggtggggacgggggcggtg
gagagggaaggttgggaggggctgcggtgccggcgggggtaggagagcggctagggcgcgagtgggaacagccgcagcggaggg
gccccggcgcggagcggggttcacgcagccgctagcgcccaggcgcctctcgccttctccttcaggtggc**gcaaaactttgtgccttgg
a**tttt**ggcaaattgttttcctcacc**gc**cacctcccgcggcttcttaa**gggcgccag**ggccgatttcgattcctctg**ccgctgcggggccg
actcccgggctttgcgctccgggctcccgggggagcgggggctcggcgggcaccaagccgctggttca**ctaagtgcgtctccgagata**
gcaggggactgtccaaagggggtg**aaagggtgctccctttattc**cccccaccaagaccacccagccg**ctttaggggatagctctgca**a
ggggagaggttcgggactgtggcgcgcactgcgcgctgcgccaggtttccg**caccaagacccctttaactc**aa**gactgcctcccgcttt
gtgt**gccccgctccag

bioRxiv preprint doi: https://doi.org/10.1101/2024.02.19.580973; this version posted February 19, 2024. The copyright holder for this preprint (which was not certified by peer review) is the author/funder. This article is a US Government work. It is not subject to copyright under 17 USC 105 and is also made available for use under a CC0 license.

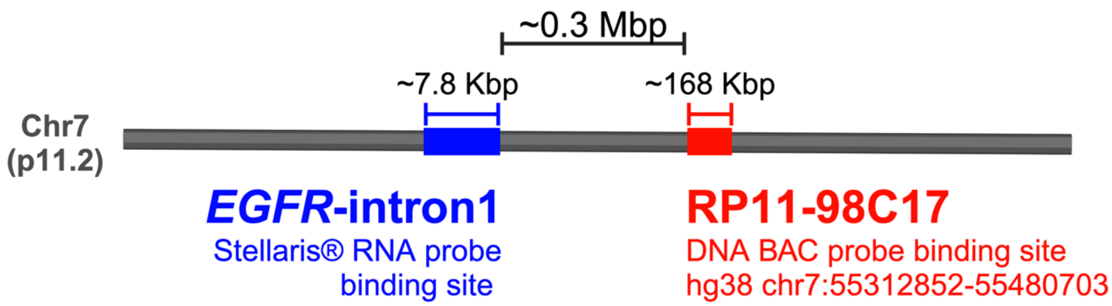

EGFR Stellaris® RNA Probes hybridization sites

(the initial 7,843 nucleotides of Intron1, hg38 chr7:55019366-55027209, strand: +, 5' to 3')

gtaagggcgtgtctcgccggctcccgcgccgcccccggatcgcgccccggaccccgcagcccgcccaaccgcgcaccggcgcaccggctcggcgcccgc
gccccccgcccgtcctttcctgtttccttgagatcagctgcgccgccgaccgggaccgcgggaggaacgg**gacgtttcgttcttcggccg**ggagagtctggggc
gggcggaggaggagacgcgtgggacaccgggctgcaggccaggcggggaacggccgccgggacctccggcgccccgaaccgctcccaactttcttccctc
actttccccgcccagctgcgcaggatcggcgtcagtgggcgaaagccgggtgctggtgggcgcctggggccggggtcccgcacgtgcgccccgcgctgtctt
cccagggcgcgacggggtcctggcgcgcacccgaggggcgggcgctgcccacccgccgagactgcactgtttagggaagctgaggaaggaacccaaaaat
acagcctcccctcggaccccgcgggacaggcggctttctgagaggacctccccgcctccgccctccgcgcaggtctcaaactgaagccggcgcccgccagcc
tggccccggcccctctccaggtccccgcgatcctcgttccccagtgtggagtcgcagcctcgacctgggagctgggagaactcgtctaccaccacctgcggctc
ccggggaggggtggtgctgg**cggcggttagtttcctcgtt**ggcaaaaggcaggtggggtccgacccgccccttgggcgcagaccccggccgctcgcctcgc
ccggtgcgc**cctcgtcttgcctatccaag**agtgccccccacctcccggggaccccagctccctcctgggcgcccgcgccgaaagccccaggctctccttcgat
ggccgcctcgcggagacgtccgggtctgctccacctgcagcccttcggtcgcgcctgggcttcgcggtggagcgggacgcggctgtccggccactgcagggg
gggatcgcgggactcttgagcggaagccccggaagcagagctcatcctggccaacaccatggtgtttcaaaatggggctcacagcaaacttctcctcaaaaccc
ggagactttctttcttggatgtctcttttgctgtttgaagaatttgagccaaccaaaatattaaacctgtcttacacacacacacacacacacacacacacacaccg
gattgctgtccctggttcaagtgtgccaagtgtgcagacagaacatgagcgagtctggcttcgtgactacc**gaccataaacccacttgaca**gggga**aacatgcc**
ttggaaggtttaattgcacaattccaaccttgagctgcgcgggttccaagagccaggcccgtact**tgctgttgatgtcattggct**tggggagttggggtttggtgc
ccagcgcggtcgttgggggaggggcaaggcatagaacagtggttcccagaccttgctgcacattggaattacctgggattaaaaaaaaaaaatcaaaacaaaa
accagtgtctggctcccgcccccagacattctgatttaattggcatggggcaagacctggacttgggatttttttaatgctcttcatgtgatctgttgggcagccagatt
tggggatcactagacggaagaaggattgttaaagtctccggagatgttacttgccaatgctaagagctct**ttgaggacatctggaattgt**tacaatattgccaaatat
aggaaagagggaaaaggtagagtgtgattccaataataaaggattccgcttttcattgaaggaac**tggtggaaaggtttcttctc**tgctgagcctgcaggcccgtc
ctgcctgcctggggtgcccgggagacgcgggcctgctccggagactgctgactgccgg**tcctgttagtcaggtgtcag**ccctgtctctgccgaagagactcttc
tctttattttaaattaaaccctcagagcaccaccaaagcatcacttttctccctccattggtgttctcattctttgatgttacttgtttgaacaccactattagtagttggagatt
tgttcctgagaaaaatataaataccacttaatttgcctgtttgtcccgcatt**cactcaaaacagaatgctcc**tgaagacaagagagagagagtaggagaacagacgct
attccattacagtaacataaaagact**ggattttcaggggcaaatta**ttaaaataggagatgagctcttttaacagaaatttgtttaaggcctgtgtctatcaaattcagt
ggattttattcaagatgcactttgtttagtgggagttttgtttg**gttctgggacatgctaactt**ctag**acttgctgctcttagaggta**atgactgccagacaccatttcat
gagtcctaatccccacattaagcataagaggtgcacactctcctcctatgggggaaactgaggtacgaagaactaaagtgactttcccacagctggtgggaggca
gacgggaaattcacaccaggggcttccaactccagatccctctctcaacttccaaactccactgccttgtccgagttctggtttcaggagatcc**aaatcaggtgtgt**
gcaaatgtctaatgtcagagctggcaaggggaaagggcccagggagccggctcatgacgatgagcctgtctgaagcttcaacgcgggctgtccggcagtctgc
attcctgcc**gagttcctcagccctctgtt**gggtcaccttccatagaggcagcttagtcctcagttcagtgagcatggagtggagactgcttgaggggtgctgagca
aagccctgcctcttacaggatgaaggtgctctccagaagggacactggaaagtattccaaggc**gagtcgaattcccaactgag**ggagctttgtggaaataagcc
cgcccagccccacttct**ggagacgttcccattcagta**gg**tccgagctgtcttaaagaga**aaccaaagtggggatattaatggtatccaaagtgagatctacccc
accctccctcctcaaaggaggtcagatcaagaaagcccaagcccggcctggcaattgggacctttcttctcactccagcccagggtgaaggtggacaagtc**actt**
tgacccttcaggcttctgagctgttgtttctgaattcagtgaatatttactgagtgcatagaatatgctagatattctgggctaaaggttgaagggggggtgagttt**taa**
gggtttctgctcttgcttccagattgctttcaaatctggaaaggacaccagtggtttgtgtgttagacccacactgccgtag**cacagaatacaagaaactgg**ctga
gagctccaataggcttttaacagtaatttctggcttcacgtatttagtttcataactcatgatttttcaaaaacttctggtttgaagaca**ccgattgccgaaagtccatt**gt
gctgcataattacacttggtccacgtgacagcactaacatgttctgaaatgtttttagaagtagtctcagcaaagatgaaggattcctccctgtttgaaaagaaaatattc

bioRxiv preprint doi: https://doi.org/10.1101/2024.02.19.580973; this version posted February 19, 2024. The copyright holder for this preprint (which was not certified by peer review) is the author/funder. This article is a US Government work. It is not subject to copyright under 17 USC 105 and is also made available for use under a CC0 license.

tttgtttttctttgatctaagctctaagactagcagctagcatctgaaactttttgacg**agagtgacaaaccaactcta**atattaaag**gcaattgatgattatgggca**
ctgaagggaaggtaaccccaggctggtgccccggaatagggatgggtcacaat**gttgaggacatttcgcctgt**tgcagaacccacctgcaacacagtgtggcc
cttgccatgtgacttgtgtgtgtgcctgtgtgtctgtgtgtgcgtgttttaattttgacttcataagtactctagttatgagcttatttaacattgggttttactaatagggtat
gtgttgagaaaatttcaaagttttagaatat**ggttcacccacatgttgctt**ccctgtaaatataatttttaaaaccagattctgggccgggcatggtggctcacctctata
atcccaaaacgttgggaggccgaggcaggcgaatcatgaagccaggagtttgagaccaggctgaccaacacggtgaaacccagtctctactaaaaatacaaaa
aaaattagctgggcgtggtggcaggtgcctgtaatcccagctactcaggaggctgaggcaggagaattgcttgaacccaggaggcagaggttgcagtgagcca
agatcgcaccattgcactccagcccgcgcgacagtgtgagactccatctcaaaaaaaaaaaaaaaaaaaacagattctgttcctcagatccattccatttttgttttcctt
tatcacttatggacatttgaaattatggtaataaacattgttagtctcagttaattattactggtttattcttgaaccactaatccatagagaatagagtgtaaatctt**aacttg**
ttcctgtaggccatccccattaaacatcatagtgttttctcattcgttctttttcgttttcctcctacaggaatgaattttctaagaaaattccagcagttggctctt**tggacg**
acatctctagattgtcctccattgggcccataggcacaagctggccagt**ttgaatttgggcaagaatcc**aggcattggaacttattcaaataactagtttgcctgta
attttcactttttcagagtcatctgataaagctttcttgctacacatttagatagatacactcaatccagttgtctagaaagttccctgagccagctgggagcaggagggg
tagttggggccaggaatattgggggt**gtgtttactgagcccctaga**aagtaagtgctagatttgacatttcaatccctgaaggccctgaagttcagtatcaaatgact
ggtcctgtggactgagcatctgtgaattgcatatgcttagagtaaatttt**actcctaccagtttcagcag**cttgctttagcaagcagtatggaaacactaacatgggg
gagtagaatttctctctctgatccaagttttatctcattctggtgggttttcaaggagagactcggagtccaagtgtcctttctgaatatatctggaacttctcattaacaaa
agactcaagttataatttaggggacaaggcacccaatgagaatgccttgcaggcagccctaagtacacctgcaattacaccattactagcgcggcagcac**acatg**
gccctgacttagtttaaataattacgtaagtcaaccatgattgtttgccctttgcatagaagggcaagtattggtacctgttacaacttaggcttttttttctttatgtttgag
ccatgatgagtgatttacactgttgcatccatatgttgagatgtaagaataaattagacttggta**attgcccttaagtgtctgga**agtcaactggggaaagagagcta
gagataataagtgtgaaacaatgtcacagaatcaatgacggaactcttccc**aggacaaaggatgacttttg**agttcagtcttt**gcctttaattctacatgggg**agg
agagc**acgtttagccacaaatggaa**gggattactcatttgagctatttggttatatgattatttccccagagaataggatgtgcagggcattacacaagcagtgcca
atagcagcaaagttcttgagagtgctagtaattcaaatggcaggaagagaaggaataaatggtaaggctacctacagttcacagagagctccatcctcactgtggc
tttggattttgtcctgtgtgaaagagaagtgactgtgaactgacatgctgtgtttggtgttttagaaagatggctgcagcagcggtttggggaatggactgcaggagtg
gcattggaaacaggaaggttcatgactattgccagagacagaggatgaagcaggagcaaggaagattcaggacaggggactccggggctgatcaggaggca
gaactggttgataagtatatgtagcagcataagaaagaaagaatcccagattgacacccaggcttctcacttggaagcctggatagatactgaatgcaatcacaaa
ggctgggaagtcaatgggactgcagggaagggaagggaagggaggagaagaggaagggcaggagggtccaatatcaatattcagcttttagatgtgttgagct
tgaagtgctcagatggagaagtccaggaggcagtagaatacggtggtccagagcacaggagagcaatgtggcttgagttgtcatttgctcacatatttccgtgtca
gttacttgtcttagatcacagaacaagttctc**ctctcacagtttcctggctc**cacctgtctcatgctc**accgtcagcatcgaaattga**gccacaccaggggtt**ctgg**
ataccagcttctctctaggtgaggctgctatag**tcagcagctgattagttgca**gttatcagcaactggtaatataatatattgtgcatataagtgtaccagaagtcat
gtttata**tattgctgcaaatactcgga**atggggatctctt**gttccctgcttaagaccaca**tcacattacttggttttgtacgctagtggctgaaccaaaaaaagtagg
agatgattttttttctttttcttaaagcagtagcttttgaaccttgaccatgctttctaaccagctgaggggcttttgaaaaagagggtgccttactgtgccccagaccag
gacaatcagtatttctggggaatggagcctggcacacacacatttcttaaagctcccttggcaattc**tgaggagtggattacatgtt**gtatgt**agctcgtaacgaa**
agaaatcttgtc**tttgctctcagacccccatt**tcttactcatctcatgagct**ccttcgagatccagaaacag**ttgcatatttcattagtaaatcagttccagagtcaca
ttttatttcacaagttagtccattaaaagtttcctgcagtgaggaaatagccagaaagaacactccacccctcctcctttttataactatagggtctggctcgacagagc
aggagcatcgccatcttggacaagcccctcattctaaagttcaccttaataaaaaactgcctaaattcaaactgcatcagcctaatggctaaggtcagcatgaccata
aaccacaaataacatctccaaccggaaacattcgaaactcctcctcgaccagagacatgctagtcccgagataaccccctccagcagggaagatgccagtctcg
ggataacctctctctggccggaaagatgcctgccccaagataaacttgcctcctcccagagatattccaaccctgccataaaacttctccctcaaacaggaacattc
caaaattctgataatctccctcaccctaaaaccaatatatactcctagtctgtaagagaaagcgctcttgaccaaaattcaccaggagtgcctcccaggttttaactaaa
gaaaacctctctttaactgccaaaaaaaaaaaagggaaaaaaaaaaagctttctgcagtggctttcagcgggcccagcatggcagcagcacctgagaacctgttgga
gatgcacactcttggaccccaccctggcctctgagtaagacactggaagggcaggccccggtctgtgcacacaagtcctcagggagattctgactgatgcatgcc
agattttgagaactgctgatatactccaggcacatcgcatgctgggatctagatacaccaagggaacaaaataactgcacttgtcctctgaggaccgacttaccttttt
ggaagggctgagaaagagagacacatacaagatcactccctgt

bioRxiv preprint doi: https://doi.org/10.1101/2024.02.19.580973; this version posted February 19, 2024. The copyright holder for this preprint (which was not certified by peer review) is the author/funder. This article is a US Government work. It is not subject to copyright under 17 USC 105 and is also made available for use under a CC0 license.

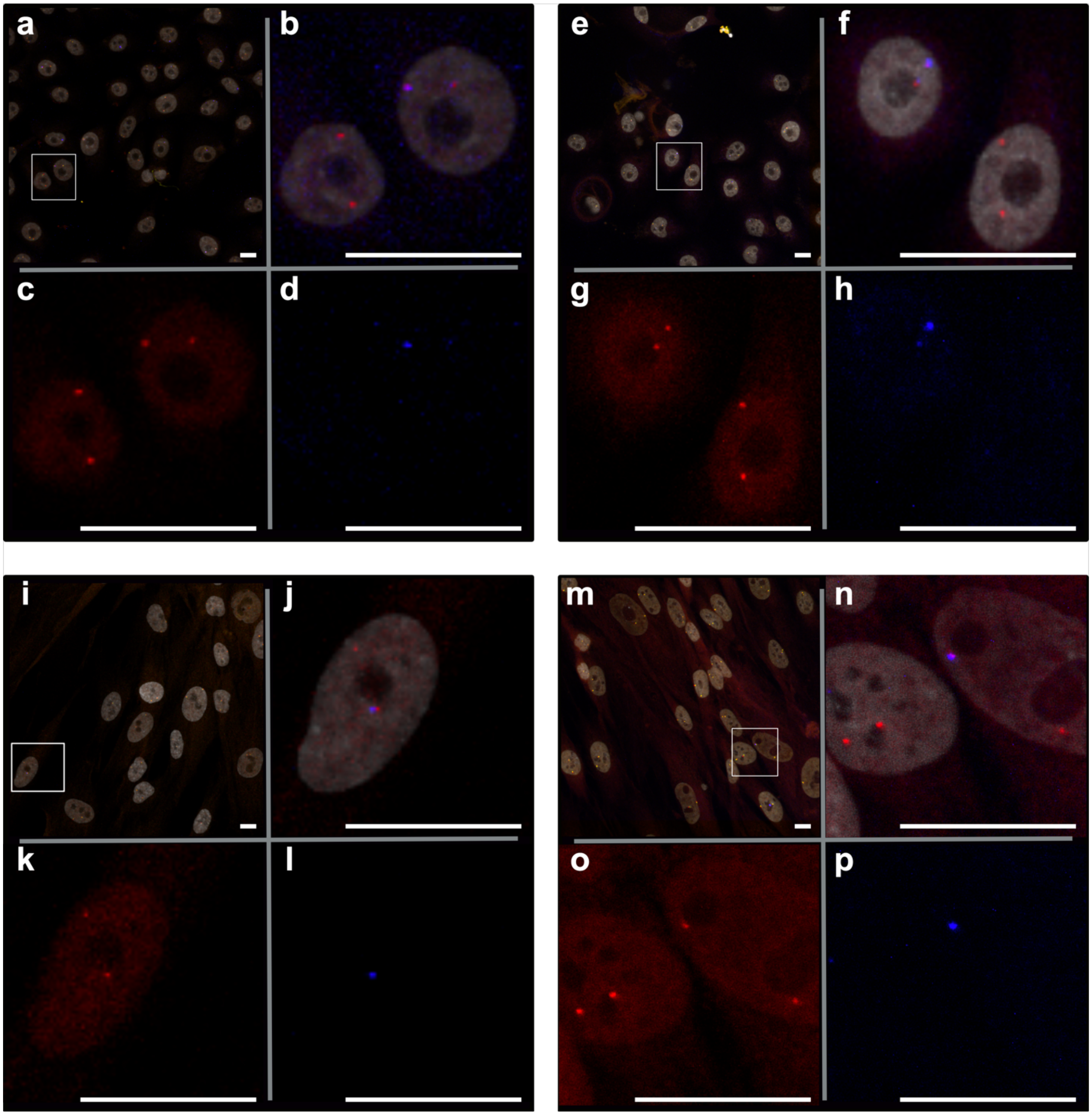

Supplementary Figure 2: Representative images of simultaneous and sequential MYC DNA/RNA HiFISH in HBEC and HFF cells. Red: DNA, blue: nascent mRNA, grey: DAPI-stained nucleus. (a-d) simultaneous FISH in HBEC. (e-h) sequential FISH in HBEC. (i-l) simultaneous FISH in HFF. (m-p) sequential FISH in HFF. Scale bars: 20 µm

bioRxiv preprint doi: https://doi.org/10.1101/2024.02.19.580973; this version posted February 19, 2024. The copyright holder for this preprint (which was not certified by peer review) is the author/funder. This article is a US Government work. It is not subject to copyright under 17 USC 105 and is also made available for use under a CC0 license.

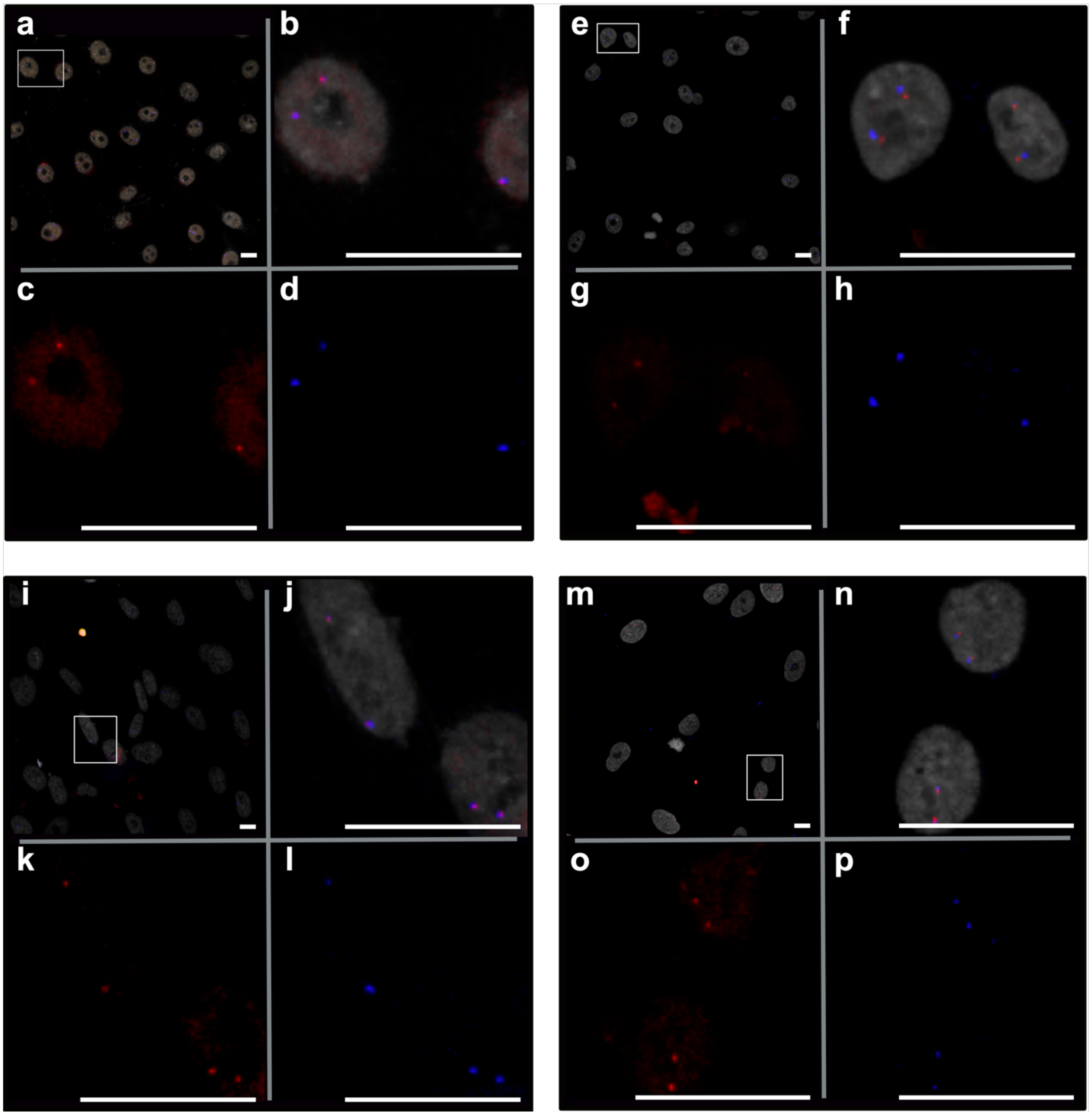

Supplementary Figure 3: Representative images of simultaneous and sequential EGFR DNA/RNA HiFISH in HBEC and HFF cells. Red: DNA, blue: nascent mRNA, grey: DAPI-stained nucleus. (a-d) simultaneous FISH in HBEC. (e-h) sequential FISH in HBEC. (i-l) simultaneous FISH in HFF. (m-p) sequential FISH in HFF. Scale bars: 20 µm

bioRxiv preprint doi: https://doi.org/10.1101/2024.02.19.580973; this version posted February 19, 2024. The copyright holder for this preprint (which was not certified by peer review) is the author/funder. This article is a US Government work. It is not subject to copyright under 17 USC 105 and is also made available for use under a CC0 license.

Supplementary Table 1 Summary of radial distance means, standard deviations, and KS test results comparing active to inactive alleles across all experimental repeats.

Condition					Radial distance			Two-sided Kolmogorov–Smirnov (KS) Test		
Gene	Cell-line	Protocol	Repeat	Activity	N Allele	Mean	SD	N Allele	p value	D Statistics
MYC	HBEC	Simultaneous	1	Active	1179	0.7	0.2	1914.00	0.37	0.04
				Inactive	735	0.7	0.2			
MYC	HBEC	Simultaneous	2	Active	125	0.6	0.2	234.00	0.33	0.12
				Inactive	109	0.7	0.2			
MYC	HBEC	Simultaneous	3	Active	1629	0.7	0.2	2978.00	0.34	0.03
				Inactive	1349	0.7	0.2			
MYC	HBEC	Sequential	1	Active	781	0.7	0.2	1380.00	0.22	0.06
				Inactive	599	0.7	0.2			
MYC	HBEC	Sequential	2	Active	154	0.7	0.2	344.00	0.42	0.10
				Inactive	190	0.8	0.2			
MYC	HFF	Simultaneous	1	Active	246	0.7	0.3	490.00	0.86	0.05
				Inactive	244	0.7	0.2			
MYC	HFF	Simultaneous	2	Active	258	0.7	0.2	652.00	0.01	0.14
				Inactive	394	0.7	0.2			
MYC	HFF	Sequential	1	Active	159	0.7	0.2	790.00	0.13	0.10
				Inactive	631	0.7	0.2			
MYC	HFF	Sequential	2	Active	73	0.7	0.2	150.00	0.28	0.16
				Inactive	77	0.8	0.2			
EGFR	HBEC	Simultaneous	1	Active	3545	0.6	0.2	4614.00	0.40	0.03
				Inactive	1069	0.6	0.2			
EGFR	HBEC	Simultaneous	2	Active	7891	0.6	0.2	9776.00	0.00	0.05
				Inactive	1885	0.6	0.2			
EGFR	HBEC	Simultaneous	3	Active	7623	0.6	0.2	9108.00	0.00	0.05
				Inactive	1485	0.6	0.2			
EGFR	HBEC	Sequential	1	Active	326	0.6	0.2	504.00	0.39	0.08
				Inactive	178	0.6	0.2			
EGFR	HBEC	Sequential	2	Active	116	0.6	0.3	166.00	0.65	0.12
				Inactive	50	0.6	0.3			
EGFR	HFF	Simultaneous	1	Active	2554	0.6	0.2	3458.00	0.74	0.03
				Inactive	904	0.6	0.2			
EGFR	HFF	Simultaneous	2	Active	1184	0.6	0.3	1902.00	0.04	0.07
				Inactive	718	0.6	0.3			
EGFR	HFF	Simultaneous	3	Active	2159	0.6	0.2	2748.00	0.14	0.05
				Inactive	589	0.6	0.2			
EGFR	HFF	Sequential	1	Active	208	0.6	0.2	368.00	0.05	0.14
				Inactive	160	0.7	0.2			

Blue font color rows indicate data corresponding to Fig. 4

bioRxiv preprint doi: https://doi.org/10.1101/2024.02.19.580973; this version posted February 19, 2024. The copyright holder for this preprint (which was not certified by peer review) is the author/funder. This article is a US Government work. It is not subject to copyright under 17 USC 105 and is also made available for use under a CC0 license.

Supplementary Table 2 List of change in radial distance mean and KS test comparing simultaneous vs. sequential FISH

Condition				Radial distance	Two-sided Kolmogorov–Smirnov (KS) Test		
**Gene**	**Cell-line**	**Activity**	**Protocol**	**Δ mean**	**N allele**	**P value**	**D statistics**
MYC	HBEC	Active	Simultaneous<br>Sequential	0.06	1960	0.00	0.23
MYC	HBEC	Inactive	Simultaneous<br>Sequential	0.06	1334	0.00	0.25
MYC	HFF	Active	Simultaneous<br>Sequential	0.06	1186	0.02	0.11
MYC	HFF	Inactive	Simultaneous<br>Sequential	0.04	1474	0.00	0.14
EGFR	HBEC	Active	Simultaneous<br>Sequential	0.03	3871	0.16	0.07
EGFR	HBEC	Inactive	Simultaneous<br>Sequential	0.04	1247	0.05	0.11
EGFR	HFF	Active	Simultaneous<br>Sequential	0.04	2762	0.06	0.10
EGFR	HFF	Inactive	Simultaneous<br>Sequential	0.09	1064	0.00	0.17
